# Oral rivaroxaban versus standard therapy for the treatment of symptomatic venous thromboembolism: a pooled analysis of the EINSTEIN-DVT and PE randomized studies

**DOI:** 10.1186/1477-9560-11-21

**Published:** 2013-09-20

**Authors:** Martin H Prins, Anthonie WA Lensing, Rupert Bauersachs, Bonno van Bellen, Henri Bounameaux, Timothy A Brighton, Alexander T Cohen, Bruce L Davidson, Hervé Decousus, Gary E Raskob, Scott D Berkowitz, Philip S Wells

**Affiliations:** 1 Maastricht University Medical Center, Maastricht, The Netherlands; 2 Bayer HealthCare AG, Wuppertal, Germany; 3 Klinikum Darmstadt, Darmstadt, Germany; 4 Hospital Beneficência Portuguesa, São Paulo, Brazil; 5 Department of Medicine, University Hospitals of Geneva, Faculty of Medicine, Geneva, Switzerland; 6 Department of Haematology, Prince of Wales Hospital, Sydney, New South Wales, Australia; 7 Department of Haematological Medicine, King’s College Hospital, London, UK; 8 University of Washington School of Medicine, Seattle, WA, USA; 9 Service de Médecine et Thérapeutique, CHU Saint-Etienne, Saint-Etienne, France; 10 University of Oklahoma Health Sciences Center, College of Public Health, Oklahoma City, OK, USA; 11 Bayer HealthCare Pharmaceuticals, Whippany, NJ, USA; 12 Department of Medicine, University of Ottawa and the Ottawa Hospital Research Institute, Ontario, Canada

**Keywords:** Rivaroxaban, Standard therapy, Venous thromboembolism, Randomized controlled trials

## Abstract

**Background:**

Standard treatment for venous thromboembolism (VTE) consists of a heparin combined with vitamin K antagonists. Direct oral anticoagulants have been investigated for acute and extended treatment of symptomatic VTE; their use could avoid parenteral treatment and/or laboratory monitoring of anticoagulant effects.

**Methods:**

A prespecified pooled analysis of the EINSTEIN-DVT and EINSTEIN-PE studies compared the efficacy and safety of rivaroxaban (15 mg twice-daily for 21 days, followed by 20 mg once-daily) with standard-therapy (enoxaparin 1.0 mg/kg twice-daily and warfarin or acenocoumarol). Patients were treated for 3, 6, or 12 months and followed for suspected recurrent VTE and bleeding. The prespecified noninferiority margin was 1.75.

**Results:**

A total of 8282 patients were enrolled; 4151 received rivaroxaban and 4131 received standard-therapy. The primary efficacy outcome occurred in 86 (2.1%) rivaroxaban-treated patients compared with 95 (2.3%) standard-therapy-treated patients (hazard ratio, 0.89; 95% confidence interval [CI], 0.66–1.19; p_noninferiority_ < 0.001). Major bleeding was observed in 40 (1.0%) and 72 (1.7%) patients in the rivaroxaban and standard-therapy groups, respectively (hazard ratio, 0.54; 95% CI, 0.37–0.79; p = 0.002). In key subgroups, including fragile patients, cancer patients, patients presenting with large clots, and those with a history of recurrent VTE, the efficacy and safety of rivaroxaban were similar compared with standard-therapy.

**Conclusion:**

The single-drug approach with rivaroxaban resulted in similar efficacy to standard-therapy and was associated with a significantly lower rate of major bleeding. Efficacy and safety results were consistent among key patient subgroups.

**Trial registration:**

EINSTEIN-PE: ClinicalTrials.gov, NCT00439777; EINSTEIN-DVT: ClinicalTrials.gov, NCT00440193.

## Introduction

Venous thromboembolism (VTE), the collective term for deep-vein thrombosis (DVT) and/or pulmonary embolism (PE), is a relatively common cardiovascular disorder. Each year, approximately one to two cases per 1000 population present with acute symptomatic VTE [1,2]. Standard therapy for these patients consists of low-molecular-weight heparin combined with a vitamin K antagonist (VKA) [3,4]. Several direct oral anticoagulants have been evaluated recently for the acute and extended treatment of symptomatic VTE [5-10], and their use could obviate the need for parenteral treatment and laboratory monitoring of the anticoagulant effect.

Two studies, EINSTEIN-DVT and EINSTEIN-PE, compared a single-drug approach using the oral, direct factor Xa inhibitor rivaroxaban with standard-therapy consisting of enoxaparin overlapping with and followed by a VKA for the treatment of DVT and/or PE [8,9]. These studies showed similar efficacy and a tendency towards a lower incidence of major bleeding with rivaroxaban; the results formed the basis for regulatory approval of rivaroxaban for acute and extended treatment of DVT and/or PE [8,9].

Both studies used an identical design, treatment regimens, outcome definitions, and adjudication processes to allow for an *a priori* pooled analysis based on individual patient data. The aim of this analysis was to provide more precise estimates of efficacy and safety, in particular the pattern of bleeding events. In addition, the analysis allowed more detailed investigation of the efficacy and safety of rivaroxaban in key clinical subgroups in which VKA therapy is associated with an increase in complications, such as in patients who are elderly or renally impaired, and in those with cancer [11-13]. Therefore, the current analysis focused on these groups of patients and, in addition, analyzed the performance of rivaroxaban in patients with previous VTE and in those presenting with a large clot burden.

## Methods

### Patients

Patients were potentially eligible if they had symptomatic DVT and/or PE. The major ineligibility criteria were: a therapeutic dose of low-molecular-weight heparin, fondaparinux, or unfractionated heparin for more than 48 hours; more than a single dose of a VKA; treatment of the current episode with thrombectomy, a vena cava filter, or fibrinolytic therapy; any contraindication listed in the local labeling of enoxaparin, warfarin, or acenocoumarol; or a creatinine clearance <30 ml/min.

### Treatment regimens

Identical rivaroxaban and standard-therapy regimens were evaluated in both trials. Patients assigned to rivaroxaban were given 15 mg orally twice-daily for 21 days, followed by 20 mg once-daily. Patients assigned to standard-therapy received enoxaparin subcutaneously at a dose of 1.0 mg/kg body weight twice-daily and either oral warfarin or acenocoumarol (international normalized ratio (INR), 2.0–3.0), started within 48 hours after randomization. Patients were treated for 3, 6, or 12 months, as determined locally.

### Follow-up and outcome assessment

Both studies used the same protocol to ascertain outcomes. Patients were followed for the intended treatment period and assessed at fixed intervals that were identical in the two treatment groups [8,9]. The prespecified efficacy outcome was symptomatic recurrent VTE, i.e. the composite of fatal or nonfatal PE or DVT [8,9]. The prespecified principal safety outcome was clinically relevant bleeding, defined as the composite of major and nonmajor clinically relevant bleeding, as described previously [8,9]. Bleeding was defined as major if it was clinically overt and associated with a decrease in hemoglobin level of ≥2.0 g/dl; if bleeding led to the transfusion of ≥2 units of red cells; or if bleeding was intracranial or retroperitoneal, occurred in another critical site, or contributed to death. Nonmajor clinically relevant bleeding was defined as overt bleeding that did not meet the criteria for major bleeding but was associated with medical intervention, unscheduled contact with a physician, interruption or discontinuation of study drug, or discomfort or impairment of activities of daily life. We also prespecified to analyze net clinical benefit, which was defined as the composite of the primary efficacy outcome and major bleeding.

### Key clinical subgroups

Efficacy and safety outcomes were separately analyzed in key prespecified subgroups of patients, i.e. fragile patients, those with cancer, patients with a previous VTE, and patients presenting with a large clot burden. Fragility was defined as one or more of the following criteria: age >75 years, calculated creatinine clearance <50 ml/min, or low body weight (≤50 kg). The presence of cancer was categorized as known active cancer at study entry or cancer diagnosed during treatment. The extent of DVT or PE was defined as (i) limited, if thrombosis was not above the popliteal vein or PE was confirmed to a single lobe involving ≤25% of the vasculature of that lobe, or (ii) extensive, if thrombosis involved the common femoral and/or iliac vein or if PE involved multiple lobes and affected >25% of the entire pulmonary vasculature. All other results for thrombosis and PE were classified as intermediate.

### Statistical analysis

The EINSTEIN-DVT and EINSTEIN-PE studies were each designed to test the hypothesis that rivaroxaban would be noninferior to standard-therapy. Each study was event-driven and required a number of events to provide 90% power using a margin of 2.0 for the upper limit of the 95% confidence interval (CI) for the estimated hazard ratio, and a two-sided alpha of 0.05. For the pooled analysis, the prespecified noninferiority margin was 1.75 [14].

The primary efficacy analysis was performed on an intention-to-treat basis with the use of a Cox proportional hazards model stratified according to the qualifying DVT or PE and the intended duration of treatment, with adjustment for the presence or absence of active cancer at baseline.

Homogeneity of the DVT and PE evaluations was tested with the Gail–Simon test of qualitative interaction [15]. Weighted absolute risk differences and their CIs were calculated using the Mantel–Haenszel asymptotic method with the weights based upon sample sizes per stratum.

To compare the pattern of major bleeding, the first event was categorized as (i) fatal, (ii) nonfatal, but in a critical organ or site, or (iii) associated with a fall in hemoglobin and/or need for transfusion. The mean time during which the INR was in the therapeutic range was calculated after the discontinuation of enoxaparin, with correction for interruptions in the administration of VKAs or the use of concomitant heparins.

## Results

### Patients

**Figure 1:**
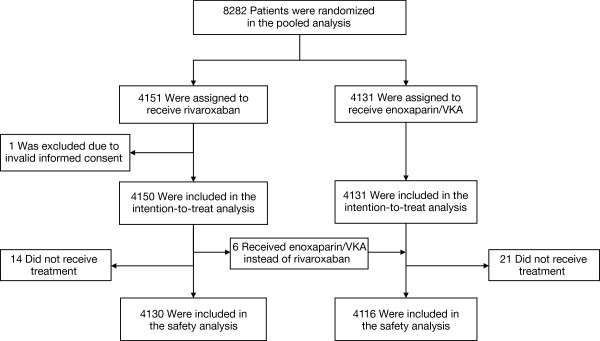
**Enrolment and outcomes. ***VKA*, Vitamin K antagonist.

Between March 2007 and March 2011, a total of 8282 patients were randomized at 314 sites in 38 countries; 4151 patients were assigned to receive rivaroxaban and 4131 were assigned to receive standard-therapy (Figure 1). The characteristics of the patients in the treatment groups were similar at baseline in both studies (Table 1) [8,9].

**Table 1:** Demographic and clinical characteristics

	**EINSTEIN-DVT**[8]		**EINSTEIN-PE**[9]		**EINSTEIN pooled**	
	**Rivaroxaban**	**Enoxaparin/VKA**	**Rivaroxaban**	**Enoxaparin/VKA**	**Rivaroxaban**	**Enoxaparin/VKA**
	**(N = 1731)**	**(N = 1718)**	**(N = 2419)**	**(N = 2413)**	**(N = 4151)**	**(N = 4131)**
Male sex–n (%)	993 (57.4)	967 (56.3)	1309 (54.1)	1247 (51.7)	2302 (55.5)	2214 (53.6)
Mean age–years ± SD	55.8 ± 16.4	56.4 ± 16.3	57.9 ± 7.3	57.5 ± 7.2	57.0 ± 17.0	57.0 ± 16.8
Risk factor associated with VTE–n (%)						
Recent surgery or trauma	338 (19.5)	335 (19.5)	415 (17.2)	398 (16.5)	753 (18.1)	733 (17.7)
Previous VTE	336 (19.4)	330 (19.2)	455 (18.8)	489 (20.3)	791 (19.1)	819 (19.8)
Active cancer	118 (6.8)	89 (5.2)	114 (4.7)	109 (4.5)	232 (5.6)	198 (4.8)
Estrogen therapy	140 (8.1)	115 (6.7)	207 (8.6)	223 (9.2)	347 (8.4)	338 (8.2)
Immobilization	265 (15.3)	260 (15.1)	384 (15.9)	380 (15.7)	649 (15.6)	640 (15.5)
Known thrombophilic condition	107 (6.2)	116 (6.8)	138 (5.7)	121 (5.0)	245 (5.9)	237 (5.7)
Unprovoked VTE	1055 (60.9)	1083 (63.0)	1566 (64.7)	1551 (64.3)	2621 (63.1)	2634 (63.8)
Duration of study treatment–days (mean [SD])*	193.6 (89.3)	187.5 (92.5)	216.3 (98.7)	214.3 (98.9)	207.6 ± 95.9	203.8 ± 97.4

*Duration of actual study treatment after randomization until end of treatment (safety population).

*SD* Standard deviation, *VKA* Vitamin K antagonist, *VTE* Venous thromboembolism.

### Treatment and follow-up

In the standard-therapy group, the median duration of enoxaparin treatment was 6 days (interquartile range, 4–9 days), and the INR at the end of enoxaparin treatment was ≥2.0 in 82.2% of patients. The mean percentage of time during which the INR was in the therapeutic range (2.0–3.0) was 61.7%; the corresponding percentages for an INR >3.0 and <2.0 were 16.0% and 22.3%, respectively. In the rivaroxaban group, adherence to therapy was ≥80% in 93.5% of patients. The mean duration of study treatment was similar in both treatment arms overall (207.6 ± 95.9 days in the rivaroxaban group and 204.0 ± 97.2 days in the standard-therapy group in the intention-to-treat population) and in prespecified patient subgroups (Table 2).

**Table 2:** Efficacy and safety outcomes and net clinical benefit in all patients and selected patient subgroups

	**Mean duration of treatment**		**Recurrent venous thromboembolism**			**Nonmajor clinically relevant and major bleeding**			**Major bleeding**			**Net clinical benefit**		
**Patient group**	**Rivaroxaban**	**Enoxaparin/VKA**	**Rivaroxaban n/N (%)**	**Enoxaparin/VKA n/N (%)**	**HR (95% CI)**	**Rivaroxaban n/N (%)**	**Enoxaparin/VKA n/N (%)**	**HR (95% CI)**	**Rivaroxaban n/N (%)**	**Enoxaparin/VKA n/N (%)**	**HR (95% CI)**	**Rivaroxaban n/N (%)**	**Enoxaparin/VKA n/N (%)**	**HR (95% CI)**
	**Days ± SD**	**Days ± SD**												
All	207.6 ± 95.9	204.0 ± 97.2	86/4150 (2.1)	95/4131 (2.3)	0.89 (0.66–1.19)	388/4130 (9.4)	412/4116 (10.0)	0.93 (0.81–1.06)	40/4130 (1.0)	72/4116 (1.7)	0.54 (0.37–0.79)	134/4150 (3.2)	169/4131 (4.1)	0.77 (0.61–0.97)
Fragile	196.8 ± 97.5	187.2 ± 102.5	21/791 (2.7)	30/782 (3.8)	0.68 (0.39–1.18)	97/788 (12.3)	109/779 (14.0)	0.85 (0.64–1.11)	10/788 (1.3)	35/779 (4.5)	0.27 (0.13–0.54)*	36/791 (4.6)	66/782 (8.4)	0.51 (0.34–0.77)^†^
Nonfragile	210.1 ± 95.4	208.0 ± 95.5	65/3359 (1.9)	65/3349 (1.9)	0.98 (0.69–1.38)	291/3342 (8.7)	303/3337 (9.1)	0.95 (0.81–1.12)	30/3342 (0.9)	37/3337 (1.1)	0.80 (0.49–1.29)	98/3359 (2.9)	103/3349 (3.1)	0.93 (0.71–1.23)
Cancer	179.0 ± 96.9	167.7 ± 103.1	16/316 (5.1)	20/281 (7.1)	0.69 (0.36–1.33)	48/316 (15.2)	44/278 (15.8)	0.93 (0.62–1.41)	9/316 (2.8)	14/278 (5.0)	0.53 (0.23–1.23)	26/316 (8.2)	37/281 (13.2)	0.60 (0.36–0.99)
No cancer	209.3 ± 95.6	205.8 ± 96.6	70/3834 (1.8)	75/3850 (1.9)	0.93 (0.67–1.29)	340/3820 (8.9)^‡^	368/3832 (9.6)^‡^	0.92 (0.79–1.06)	31/3820 (0.8)^‡^	58/3832 (1.5)^‡^	0.53 (0.34–0.82)	108/3834 (2.8)	132/3850 (3.4)	0.81 (0.63–1.05)
Previous DVT/PE	259.0 ± 102.0	252.6 ± 107.1	11/791 (1.4)	25/819 (3.1)	0.45 (0.22–0.91)^§^	70/788 (8.9)	91/813 (11.2)	0.77 (0.57–1.06)	7/788 (0.9)	14/813 (1.7)	0.51 (0.21–1.27)	20/791 (2.5)	39/819 (4.8)	0.52 (0.31–0.90)
No previous DVT/PE	195.4 ± 90.3	192.0 ± 90.8	75/3359 (2.2)	70/3312 (2.1)	1.04 (0.75–1.45)	318/3342 (9.5)	321/3303 (9.7)	0.96 (0.82–1.12)	33/3342 (1.0)	58/3303 (1.8)	0.54 (0.35–0.83)	114/3359 (3.4)	130/3312 (3.9)	0.84 (0.66–1.08)
Clot burden (limited)^¶^	197.6 ± 93.2	197.1 ± 94.1	10/799 (1.3)	19/815 (2.3)	0.51 (0.24–1.10)	73/796 (9.2)	76/813 (9.3)	0.97 (0.70–1.34)	8/796 (1.0)	11/813 (1.4)	0.75 (0.30–1.87)	20/799 (2.5)	30/815 (3.7)	0.65 (0.37–1.16)
Clot burden (intermediate)^¶^	215.0 ± 94.5	206.1 ± 96.4	41/1873 (2.2)	49/1881 (2.6)	0.82 (0.54–1.24)	181/1864 (9.7)	189/1876 (10.1)	0.95 (0.78–1.17)	20/1864 (1.1)	32/1876 (1.7)	0.62 (0.36–1.09)	65/1873 (3.5)	81/1881 (4.3)	0.79 (0.57–1.10)
Clot burden (extensive)^¶^	205.8 ± 97.8	205.2 ± 100.0	35/1364 (2.6)	26/1327 (2.0)	1.29 (0.78–2.15)	126/1359 (9.3)	134/1326 (10.1)	0.90 (0.71–1.15)	11/1359 (0.8)	28/1326 (2.1)	0.36 (0.18–0.73)	48/1364 (3.5)	56/1327 (4.2)	0.81 (0.55–1.19)

*For major bleeding, the p-value for the treatment group × fragility interaction was 0.011, suggesting heterogeneity. ^†^For net clinical benefit, the p-value for the treatment group × fragility interaction was 0.017. ^‡^In this analysis, six DVT patients were randomized to rivaroxaban but received comparator. ^§^For recurrent VTE, the p-value for the treatment group × previous DVT/PE was 0.032. For all other outcomes and analyses the interactions between treatment group and the subgroups of fragility [yes/no], baseline cancer [yes/no], previous DVT/PE [yes/no], clot burden for outcomes [limited/intermediate/extensive]), the p_interactions_ were larger than 0.05 (nonsignificant) calculated by the corresponding Cox proportional hazards models. ^¶^In the clot burden analysis, a total of 71 patients in the rivaroxaban group and 68 patients in the enoxaparin/VKA group had missing data. A further 43 and 40 patients in the rivaroxaban and enoxaparin/VKA groups, respectively, did not have confirmed DVT and PE.

*CI* Confidence interval, *DVT* Deep vein thrombosis, *HR* Hazard ratio, *PE* Pulmonary embolism, *VKA* Vitamin K antagonist.

### Clinical outcomes

**Figure 2:**
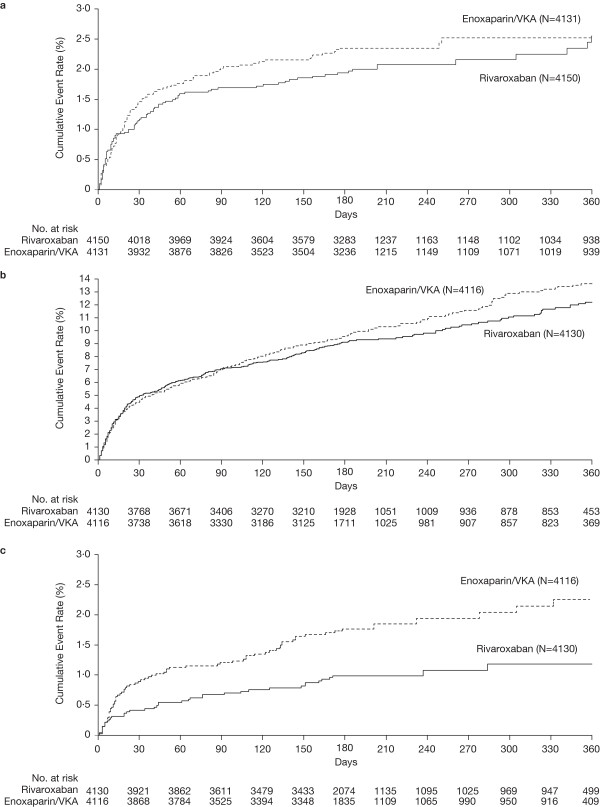
**Kaplan–Meier curves. a**. Primary efficacy outcome, **b**. Principal safety outcome and **c**. Major bleeding. *VKA*, Vitamin K antagonist.

The primary efficacy outcome occurred in 86 (2.1%) rivaroxaban patients compared with 95 (2.3%) standard-therapy patients; hazard ratio, 0.89 (95% CI, 0.66 to 1.19); one-sided p < 0.001 for the noninferiority margin of 1.75 and two-sided p = 0.41 for superiority; and absolute risk reduction of 0.2% in favor of rivaroxaban (95% CI, –0.4% to 0.9%) (Table 3; Figure 2). No heterogeneity between the DVT and PE trial results was present (Gail–Simon test of qualitative interaction, p = 0.29).

**Table 3:** Clinical outcomes

	**EINSTEIN-DVT**		**EINSTEIN-PE**		**EINSTEIN pooled**	
**Outcome**	**Rivaroxaban**	**Enoxaparin/VKA**	**Rivaroxaban**	**Enoxaparin/VKA**	**Rivaroxaban**	**Enoxaparin/VKA**
**Efficacy**						
Intention-to-treat population–N	1731	1718	2419	2413	4150	4131
First recurrent VTE–n (%)	36 (2.1)	51 (3.0)	50 (2.1)	44 (1.8)	86 (2.1)	95 (2.3)
Fatal PE	0	0	2	1	2 (<0.1)	1 (<0.1)
Death (PE cannot be excluded)	2	6	8	5	10 (0.2)	11 (0.3)
DVT and PE	1	0	0	2	1 (<0.1)	2 (<0.1)
DVT only	14	28	18	17	32 (0.8)	45 (1.1)
PE only	19	17	22	19	41 (1.0)	36 (0.9)
Death from any cause–n (%)	38 (2.2)	49 (2.9)	58 (2.4)	50 (2.1)	96 (2.3)	99 (2.4)
PE/PE not ruled out	4	6	11	7	15 (0.4)	13 (0.3)
Bleeding	1	5	5	4	6 (0.1)	9 (0.2)
Cardiovascular	2	4	10	3	12 (0.3)	7 (0.2)
Other	31	34	32	36	63 (1.5)	70 (1.7)
**Safety**						
N	1718	1711	2412	2405	4130	4116
First major plus nonmajor clinically relevant bleeding event–n (%)	139 (8.1)	138 (8.1)	249 (10.3)	274 (11.4)	388 (9.4)	412 (10.0)
First major bleeding event–n (%)						
Any	14 (0.8)	20 (1.2)	26 (1.1)	52 (2.2)	40 (1.0)	72 (1.7)
Fatal bleeding	1 (<0.1)	5 (0.3)	2 (<0.1)	3 (0.1)	3 (<0.1)	8 (0.2)
Retroperitoneal	0	0	0	1 (<0.1)	0	1 (<0.1)
Intracranial	0	2 (0.1)	2 (<0.1)	2 (<0.1)	2 (<0.1)	4 (<0.1)
GI/thorax	1 (<0.1)	3 (0.2)	0	0	1 (<0.1)	3 (<0.1)
Nonfatal in a critical site*	3 (0.2)	3 (0.2)	7 (0.3)	26 (1.1)	10 (0.2)	29 (0.7)
Retroperitoneal	0	1 (<0.1)	1 (<0.1)	6 (0.2)	1 (<0.1)	7 (0.2)
Intracranial	2 (0.1)	0	1 (<0.1)	9 (0.4)	3 (<0.1)	9 (0.2)
Intraocular	1 (<0.1)	0	2 (<0.1)	2 (<0.1)	3 (<0.1)	2 (<0.1)
Pericardial	–	–	0	2 (<0.1)	0	2 (<0.1)
Intra-articular	0	1 (<0.1)	0	3 (0.1)	0	4 (<0.1)
Adrenal/pulmonary/abdominal	–	–	3 (<0.1)	2 (<0.1)	3 (<0.1)	2 (<0.1)
Nonfatal, noncritical site but associated with a fall in hemoglobin ≥2 g/dl and/or transfusions ≥2 units	10 (0.6)	12 (0.7)	17 (0.7)	25 (1.0)	27 (0.7)	37 (0.9)
Nonmajor clinically relevant bleeding–n (%)^†^	126 (7.3)	119 (7.0)	228 (9.5)	227 (9.4)	354 (8.6)	346 (8.4)

*Columns may fail to sum because of additional bleeding events at other sites. ^†^Refers to all nonmajor clinically relevant bleeding events, not just the first event.

*DVT* Deep-vein thrombosis, *GI* Gastrointestinal, *PE* Pulmonary embolism, *VKA* Vitamin K antagonist, *VTE* Venous thromboembolism.

By day 21, at the end of twice-daily rivaroxaban administration, the primary efficacy outcome had occurred in 39 (0.9%) rivaroxaban patients and in 50 (1.2%) standard-therapy patients.

The principal safety outcome, a first major or nonmajor clinically relevant bleeding event, occurred in 388 (9.4%) rivaroxaban patients compared with 412 patients (10.0%) in the standard-therapy group; hazard ratio, 0.93 (95% CI, 0.81 to 1.06) and absolute risk reduction, 0.6% (95% CI, –0.7% to 1.9%; p = 0.27) (Table 3, Figure 2). A first major bleeding was observed in 40 (1.0%) rivaroxaban patients and in 72 (1.7%) standard-therapy patients (hazard ratio, 0.54; 95% CI, 0.37 to 0.79; p = 0.002; absolute risk reduction, 0.8% in favor of rivaroxaban; 95% CI, 0.3% to 1.3%; p = 0.002). The difference was determined primarily by the reductions in the numbers of intracranial (five vs. 13), retroperitoneal (one vs. eight) (Table 3), and gastrointestinal (including rectal and anal) bleeding events (15 vs. 26). By day 21, major bleeding had occurred in 16 (0.4%) rivaroxaban patients and in 33 (0.8%) standard-therapy patients. The composite of recurrent VTE and major bleeding (net clinical benefit) was significantly improved in patients treated with rivaroxaban, occurring in 134 of 4150 patients in the rivaroxaban group compared with 169 of 4131 patients in the standard-therapy group (Table 2, hazard ratio, 0.77; 95% CI, 0.61 to 0.97).

### Fragile patients

**Figure 3:**
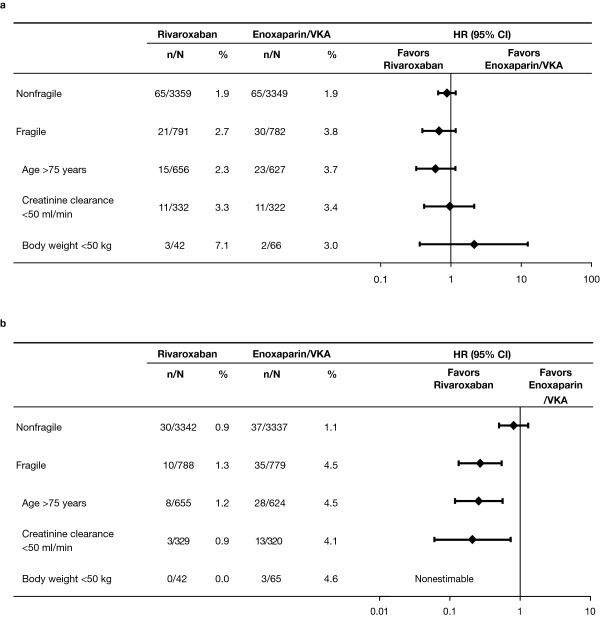
**Efficacy and safety outcomes in fragile patients and subgroups. a**. Primary efficacy. **b**. Major bleeding. *CI*, Confidence interval; *HR*, Hazard ratio; *VKA*, Vitamin K antagonist.

In total, 1573 patients (19.0%) were categorized as fragile because of age (n = 1279), moderate or severe renal impairment (n = 649), or low body weight (n = 107). Rates of recurrent VTE were higher in fragile patients than in nonfragile patients (rivaroxaban 2.7% vs. 1.9%; standard-therapy 3.8% vs. 1.9%, respectively). In fragile and nonfragile patients, the difference in recurrence rates between the rivaroxaban and standard-therapy groups was not statistically significant (Table 2). In contrast, a statistically significant difference for major bleeding in favor of rivaroxaban (1.3%) compared with standard-therapy (4.5%) was observed in fragile patients (hazard ratio, 0.27; 95% CI, 0.13 to 0.54; absolute risk reduction, 3.2%; 95% CI 1.6% to 4.9%), whereas such differences were not seen in nonfragile patients (0.9% vs. 1.1%; p = 0.01 for interaction). The effects on recurrent VTE and bleeding were consistent across the individual components that defined the fragile population (Figure 3). In fragile patients, the net clinical benefit (hazard ratio, 0.51; 95% CI, 0.34 to 0.77) was statistically significantly more favorable in rivaroxaban patients (Table 2).

### Active cancer

A total of 430 patients (5.2%) had known active cancer at baseline, whereas 167 patients (2.0%) were diagnosed with cancer during the study. In those with cancer at baseline, recurrent VTE was observed in six (2.6%) of 232 rivaroxaban patients and in eight (4.0%) of 198 standard-therapy patients, whereas major bleeding occurred in six (2.6%) of 232 rivaroxaban patients and in eight (4.1%) of 196 standard-therapy patients. Among the 167 patients diagnosed with cancer during the study, recurrent VTE was diagnosed in 10 (11.9%) of 84 rivaroxaban patients and in 12 (14.5%) of 83 standard-therapy patients. In this group, major bleeding occurred in three (3.6%) and six (7.3%) patients, respectively.

In patients with cancer, recurrent VTE occurred in 16 (5.1%) of 316 rivaroxaban patients and in 20 (7.1%) of 281 standard-therapy patients (hazard ratio, 0.69; 95% CI, 0.36 to 1.33; absolute risk reduction, 2.0%; 95% CI –1.8% to 5.8%). Of the 16 events in the rivaroxaban group, 13 occurred while patients were still on assigned treatment; the corresponding numbers in the standard-therapy group were 20 and 16 events. Major bleeding occurred in nine patients (2.8%) and in 14 patients (5.0%), respectively (hazard ratio, 0.53; 95% CI, 0.23 to 1.23; absolute risk reduction, 2.2%; 95% CI, –1.0 to 5.4%). In patients with cancer, the net clinical benefit (hazard ratio, 0.60; 95% CI, 0.36 to 0.99) was statistically significantly more favorable in rivaroxaban patients (Table 2).

### Extent of deep-vein thrombosis and pulmonary embolism at study entry

Among patients with limited thrombosis, recurrent VTE occurred in 10 (1.3%) of 799 rivaroxaban patients compared with 19 (2.3%) of 815 standard-therapy patients. In those with an intermediate clot burden, these incidences were 2.2% (41 of 1873) and 2.6% (49 of 1881), respectively. In patients with extensive clots, recurrent VTE was found in 2.6% (35 of 1364) of rivaroxaban patients and in 2.0% (26 of 1327) of standard-therapy patients (Table 2).

### Previous venous thromboembolism

Of the 1610 patients who had a previous venous thromboembolic event at study entry, 11 of 791 patients (1.4%) in the rivaroxaban group and 25 of 819 patients (3.1%) in the standard-therapy group developed a recurrent venous thromboembolic event (hazard ratio, 0.45; 95% CI, 0.22 to 0.91). This compared with 75 of 3359 patients (2.2%) in the rivaroxaban group and 70 of 3312 patients (2.1%) in the standard-therapy group who presented with a first episode of VTE (hazard ratio, 1.04; 95% CI, 0.75 to 1.45). In patients with previous VTE, the net clinical benefit (hazard ratio, 0.52; 95% CI, 0.31 to 0.90) was statistically significantly more favorable in patients treated with rivaroxaban (Table 2).

## Discussion

This prespecified pooled analysis of two large, randomized studies evaluated the efficacy and safety of a single-drug approach with oral, fixed-dose rivaroxaban versus the combination of subcutaneous enoxaparin and INR-titrated VKA therapy in patients with acute symptomatic VTE. We found that rivaroxaban had similar efficacy as standard-therapy and was associated with a significantly lower rate of major bleeding. Efficacy and safety results were consistent among key patient subgroups. Overall, the rates of recurrent VTE and major bleeding were low in the standard-therapy group and were similar to those in contemporary clinical studies [5,10,16,17].

Some limitations of our study should be noted. Firstly, we used an open-label design that could have biased assessment of outcomes. Nevertheless, efforts were made to limit investigator bias, including the requirement to use objective and validated tests to confirm suspected recurrent VTE and the use of an independent committee, whose members were blinded to treatment assignment, to adjudicate outcome events. Secondly, in the standard-therapy arm the choice of VKA was limited to acenocoumarol and warfarin, which might not have been the VKA of choice for some participating centers. However, INR monitoring was intensive and required assessment at least once a month. The time patients spent in the target therapeutic range (INR, 2.0–3.0) was in line with that achieved in contemporary studies [18]. Therefore, we believe the results obtained in this large, global study, with limited exclusion criteria, are valid and can be generalized to typical patients with DVT and/or PE.

Pooling the results of EINSTEIN-DVT and EINSTEIN-PE enabled us to derive clinically relevant results for important subgroups of patients. Large clots and a history of (multiple episodes of) VTE are conditions that are of special concern to clinicians. Here, the efficacy of rivaroxaban was similar in patients presenting with small, intermediate, or extensive DVT or PE and was similar to standard-therapy. Likewise, the efficacy of rivaroxaban was similar both in patients presenting with a first episode of VTE and in those with a history of one or more episodes of VTE.

Bleeding is the most common complication of anticoagulant treatment; in this analysis, it occurred as often in patients receiving rivaroxaban as in patients receiving standard-therapy. However, major bleeding, the most worrying complication, was statistically significantly less frequent in patients treated with rivaroxaban. This reduction was seen mainly in fatal and nonfatal critical-site bleeding, such as intracranial and retroperitoneal bleeding. It is well known that VKA therapy is a challenge in patients who are elderly, have impaired renal function, are frail, or have cancer. Particularly in these patients, rivaroxaban yielded favorable clinical results. In fragile patients, the incidence of major bleeding was reduced from 4.5% with standard-therapy to 1.3% with rivaroxaban. This translates into averting one major bleeding event for every 31 fragile patients treated with rivaroxaban rather than standard-therapy, and the risk of recurrent VTE remained similar. In patients with cancer, the incidence of both bleeding and recurrent VTE tended to be lower, resulting in a favorable benefit–risk profile. However, selective inclusion of patients with cancer might have occurred: patients with a life expectancy of <3 months were excluded, and, according to guidelines, long-term subcutaneous low-molecular-weight heparin is the treatment of choice for patients with VTE and cancer [19]. In addition, patients diagnosed with cancer after randomization were retrospectively reclassified to the cancer group. However, unless rivaroxaban or standard-therapy could have differential effects on a new diagnosis of cancer during the trial, the principle of randomization was maintained using an intention-to-treat approach to the analysis. Therefore, we believe that in patients with cancer who develop VTE, rivaroxaban may be considered as an alternative in those cases in which the attending physician would have given standard-therapy rather than long-term low-molecular-weight heparin. Based on the observed results in patients with cancer, a head-to-head comparison of rivaroxaban with long-term low-molecular-weight heparin seems warranted.

## Conclusions

The prespecified, individual, patient data meta-analysis presented here included more than 8000 patients and shows that rivaroxaban can be used as a single-drug approach for the treatment of acute symptomatic DVT and/or PE. Particularly in those patients in whom VKA therapy is associated with an increase in complications, use of rivaroxaban resulted in an important safety advantage.

## Appendix

Members of the EINSTEIN Investigators are listed in the supplementary information (Additional file 1).

## Abbreviations

DVT: Deep-vein thrombosis; INR: International normalized ratio; PE: Pulmonary embolism; VKA: Vitamin K antagonist; VTE: Venous thromboembolism.

## Competing interests

Bayer HealthCare and Janssen Pharmaceuticals supported this study, were involved in the design of the trial, and collected and analyzed the data. MHP has received research support and honoraria, and participated in advisory boards for Bayer HealthCare, Sanofi-aventis, Boehringer Ingelheim, GlaxoSmithKline, Daiichi Sankyo, LeoPharma, ThromboGenics, and Pfizer. AWAL and SDB are employees of Bayer HealthCare Pharmaceuticals. RB has received research support from Bayer HealthCare Pharmaceuticals, Bristol-Myers Squibb, Boehringer and Daiichi Sankyo, and participated in scientific advisory boards organized by all of the former companies. BvB has been an Advisory Board member for Bayer HealthCare Pharmaceuticals. HB has received research grant support from the Swiss National Foundation, Daiichi Sankyo, and Bayer Schering Pharma as well as honoraria for lectures or consultancy from Pfizer and Bayer Schering Pharma. TAB has received consultancy fees from Pfizer Australia, GlaxoSmithKline Australia, Amgen Australia, Boehringer Ingelheim, Bayer Australia, and Daiichi Sankyo. ATC has served on advisory boards for Bayer, Bristol-Myers Squibb, Daiichi Sankyo, Johnson & Johnson, Pfizer, Portola, and Sanofi, and has received consulting fees, lecture fees, support for manuscript preparation, and payment for the development of educational presentations from Astellas, AstraZeneca, Bayer, Boehringer Ingelheim, and Bristol-Myers Squibb. BLD is a member of steering committees for Bayer and Daiichi Sankyo. HD is a member of the study management and co-ordination Committee for the EINSTEIN study program; has received grants from Bayer Schering Pharma; and has participated as a board member for Daiichi Sankyo, Sanofi-aventis, and GlaxoSmithKline. GER has received consulting fees or honoraria from ICTOM/Bayer HealthCare, Takeda R&D, and Quintiles. PSW has received research support from Bristol-Myers Squibb, Pfizer; has participated on scientific advisory boards for Bayer Schering Pharma, Pfizer, and Boehringer Ingelheim; and has received honoraria from Bayer Schering Pharma, Pfizer, and Biomerieux.

## Authors’ contributions

MHP and AWAL created the initial draft version of this manuscript. All authors made critical revisions of the manuscript for important intellectual content, approved the final version of the manuscript for submission, and contributed to the study concept, design and implementation.

## Authors’ information

The EINSTEIN study group Writing Committee:

Martin H Prins, Anthonie WA Lensing, Rupert Bauersachs, Bonno van Bellen, Henri Bounameaux, Timothy A Brighton, Alexander T Cohen, Bruce L Davidson, Hervé Decousus, Gary E Raskob, Scott D Berkowitz, Philip S Wells

The members of the Writing Committee take responsibility for the content and integrity of this article.

## Supplementary Material

Additional file 1 Members of the EINSTEIN Investigators. Click here for file
