# Supplementary material for: Oral rivaroxaban versus standard therapy for the treatment of symptomatic venous thromboembolism: a pooled analysis of the EINSTEIN-DVT and PE randomized studies
Source: Thromb J. 2013 Sep 20;11:21. doi: 10.1186/1477-9560-11-21 (PMC3850944; doi:10.1186/1477-9560-11-21)
Supplement: Additional file 1 — Members of the EINSTEIN Investigators. [file 1477-9560-11-21-S1.docx]

Supplementary information

EINSTEIN study group

*Writing group* – MH Prins, AWA Lensing, R Bauersachs, B van Bellen, H Bounameaux, TA Brighton, AT Cohen, BL Davidson, H Decousus, GE Raskob, SD Berkowitz, PS Wells.

*Steering committee –* Executive committee: MH Prins, G Agnelli, SD Berkowitz, H Bounameaux, HR Büller, AT Cohen, A Gallus, AWA Lensing, F Misselwitz, L Haskell, G Peters, GE Raskob, S Schellong. Study management and co-ordination committee: R Bauersachs, B van Bellen, Z Boda, L Borris, B Brenner, TA Brighton, J Chlumsky, BL Davidson, H Decousus, H Eriksson, B Jacobson, AJ Kakkar, YL Kwong, LH Lee, K Meijer (J van der Meer), E Minar, M Monreal, F Piovella, PM Sandset, M Smith, W Tomkowski, P Verhamme, Y Wang, PS Wells.

*Central independent adjudication committee –* D Brandjes, MM Gillavry, HM Otten, MH Prins.

*Data safety monitoring board* – A Carlsson, S Laporte, S Schulman

*Sponsor (Bayer HealthCare study management) –* AWA Lensing, E Muehlhofer, J Whatton, M Trajanovic, K Mueller, C Kim, M Gebel, A Benson, AF Pap, J Godrie, A Horvat-Broecker, G Spadari, C Peters-Wulf, D Kubitza, W Mueck.

*Investigators* – (number of patients randomised and number of sites in each country is in parentheses)*. Andorra* (2 patients, 1 site) *–* J Roig; *Australia* (768 patients, 24 sites) – P Coughlin, H Salem, B Chong, S Ramanathan, C Archis, P Carroll, R Geraghty, G Connors, R Baker, D Coghlan, A Gallus, P Crispin, T Eng Gan, H Tran, C Coleman, A McCann, H Gibbs, S McRae, A Khalafallah, M Leyden, D Leyden, C Sturtz, P Crispin, C Ward, J Curnow, A Bianchi, B Richards, M Leahy, T Brighton, P Blombery, P Campbell, M Dean, R Soni, D Jackson, C Denaro, P Kubler; *Austria* (323 patients, 6 sites) – M Hirschl, M Baghestanian, B Erdogmus, E Samaha, M Nikoupayan-Mofrad, A Weltermann, H Drexel, R Mathies, E Pilger, M Brodmann, W Sturm, R Kirchmair, P Marschang; *Belgium* (272 patients, 14 sites) – R Vossaert, P Hainaut, L Pothen, S Motte, P Verhamme, K Peerlinck, M Delcroix, R Verhaeghe, P Verstraeten, V Smet, M. Gustin, J Vandekerkhof, P Vleeschauwer, H Schroë, J de Leersnyder, J Demelenne, M Buche, F Vermassen, M Sprynger; *Brazil* (66 patients, 8 sites) – B van Bellen, R Moreira, J Costa, R Sacilotto, J Silvestre, W Yoshida, M Panico, C Porto, N Wolosker; *Canada* (256 patients, 5 sites) – M Kovacs, A LazoLangner, P Wells, M Rodger, M Carrier, T Wong, R Selby, W Geerts, J Blondal; *China* (439 patients, 25 sites) – Z Chen, J Zhang, Y Wang, C Liu, R Chen, J Bi, Y Dong, C Xie, W Guo, S Long, D Wu, C Wang, YH Yang, L Zhihong, K Ying, Y Ma, K Ying, Y Shao, X Li , B Jin, S Liu, J Xu, Z-C Jing, J He, Z Jing, Z Liu, Z Jing; *Czech Republic* (455 patients, 8 sites) – K Kovářová, J Gilík, J Dosál, R Spacek, R Urbanova, I Podpera, M Podperova, F Patek, S Jirat, M Vitovec, J Chlumsky, D Hola, P Matoška, E Mandakova; *Denmark* (37 patients, 3 sites) – S Husted, C Tuxen, P Hildebrandt, H Nielsen; *Estonia* (8 patients, 1 site) – K Sukles; *Finland* (10 patients, 2 sites) – K Pietila, M Vesanen; *France* (1416 patients, 43 sites) – H Decousus, S Aquilanti, A Rifaï, G Pernod, B Imbert, P Lacroix, P Mismetti, A Buchmuller, S Accassat, J Schmidt, N Breuil, S Heuser, D Brisot, C Brousse, P Tarodo, I Quere, D Mottier, E Le Moigne, F Couturaud, D Stephan, A Achkar, H Boccalon, TA Szwebel, G Simoneau, JF Bergamnn, JL Lorenzini, A Trinh-Duc, A Gaillardou, I Mahe, L Alavoine, B Crestani, A Elias, M Elias, J Emmerich, N Meneveau, F Schiele, PM Roy, JM de Boisjolly-Bonnefoi, T Guérin, P Beaka, C Grange, T Fassier, MA Sevestre, D Vital­Durand, J Constans, Y Benhamou, D Elkouri, M Dary, E de Maistre, N Falvo, I Quere, J P Galanaud, A Queguiner, C Le Jeunne, D Wahl, B Agraou, E Ferrari, G Meyer, O Sanchez, B Planquette, G Potel, F Parent, C Boulon, T Berremili, MA Sevestre, A Bura, C Amid-Lacombe, J Malloizel; *Germany* (601 patients, 28 sites) – D Franke, S Schellong, L Pomper, R Frommhold, W Petermann, J Beyer-Westendorf, K Halbritter, S Werth, C Diehm, H Lawall, P Baron Von Bilderling, U Hoffmann, M Czihal, C Hasslacher, H Landgraf, Th Horacek, R Bauersachs, E Lindhoff-Last, F Heckmann, W Mondorf, C Espinola-Klein, G Weisser, HU Haering, A Kieback, P Kuhlencordt, E Giannitsis, J Ranft, M Schwaiblmair, Th Berghaus, M Ibe, A Bauer, N Malyar, B Taute, B Eifrig, J Potratz, T Herrmann, M Röcken; *Hong Kong* (7 patients, 2 sites) – YK Lau, E Tse; *Hungary* (331 patients, 10 sites) – A Kovács, Gy Sipos, K Farkas, E Kolossváry, M Gurzó, G Lupkovics, Z Boda, Zs Olah, E Kis, M Riba, A Landi, Zs Pecsvarady; *India* (50 patients, 4 sites) – R Parakh, P Ramakrishna, S Sudhindran, D Kamerkar; *Indonesia* (79 patients, 4 sites) – R Sembiring, C Soeharti, K Tambunan, R Sumantri; *Ireland* (2 patients, 1 site) – J Barton; *Israel*(311 patients, 12 sites) – D Zeltser, O Rogowski, A Steinvil, M Elias, L Goldstein, R Hoffman, M Lishner, A Elis, D Gavish, O Hussein, G Lugassy, D Varon, A Inbal, D Zisman, L Schliamser, M Haran; *Italy* (348 patients, 15 sites) – A Ghirarduzzi, M Iotti, M Barone, C Beltrametti, D Imberti, P Prandoni, L Spiezia, E Porreca, M Di Nisio, I Martinelli, S Siragusa, W Ageno, G Ambrosio, G Palareti, A D’Angelo, R Quintavalla, C Lodigiani, M Giorgi Pierfranceschi, M Cattaneo; *Korea*(21 patients, 4 sites) – K Park, JW Ha, HS Kim, WH Cho; *Latvia* (4 patients, 1 site) – I Sime; *Lithuania* (10 patients, 2 sites) – S Miliauskas, R Petrauskiene; *Malaysia* (12 patients, 2 sites) – J Sathar; *The Netherlands* (643 patients, 7 sites) – K Meijer, J van der Meer, A Mäkelburg, M Knol, V Tichelaar, M van Marwijk Kooy, J Coenen, A Beeker, R Komdeur, P Kamphuisen, R Douma, M De Groot, H ten Cate; *New Zealand* (203 patients, 6 sites) – P Ockelford, L Young, D Simpson, S Chunilal, P Harper, E Knottenbelt, G Royle, R Beasley, M Smith; *Norway* (86 patients, 4 sites) – P Quist‐Paulsen, PM Sandset, W Ghanima, S Foyn, A Tveit; *Philippines* (33 patients, 2 sites) – MT Abola, D Roxas; *Poland* (125 patients, 13 sites) – P Gorski, P Chęciński, R Adamiec, J Lewczuk, M Nowak, J Kloczko, W Witkiewicz, W Tomkowski, J Wronski , J Musial, G Oszkinis, J Strzelczyk, P Szyber, M Jackowski; *Singapore* (21 patients, 2 sites) – HJ Ng, C Tay; *South Africa* (354 patients, 12 sites) – JH Jansen van Rensburg, JHR Becker, R Isaacs, B Bloy, R Allie, F Eckstein, B Jacobson, D Adler, H Siebert, R Siebert, L van Zyl, N Wright, J van Marle, G Ellis, S Schmidt; *Spain*(110 patients, 9 sites) – F Del Campo, M Carrera, I Diego, D Jiménez, P Roman Sanchez, J Villalta, F. García‐Bragado, J Sánchez-Álvarez, M Redondo, J Fontcuberta; *Sweden* (114 patients, 6 sites) – H Eriksson, M Villegas-Scivetti, L Lapidus, E Ottosson, J Aagesen, T Jonson, H Tygesen, A Själander; *Switzerland* (108 patients, 7 sites) – I Baumgartner, L Mazzolai, H Bounameaux, M Righini, D Hayoz, D Periard, M Banyai, M Heidemann, U Frank, W Reinhardt, J Dorffler‐Melly, L Asmis; *Taiwan* (19 patients, 6 sites) – WT Chang, KM Chiu, KY Wang, Z-C Weng, W Tsai, TJ Yu; *Thailand* (31 patients, 3 sites) – C Pothirat, P Angchaisuksiri, P Rojnuckarin; *United Kingdom* (51 patients, 5 sites) – J Luckit, BJ Hunt, T Nokes, A Cohen, J Solis, M Sekhar; *United States* (557 patients, 26 sites) – D Feinbloom, J Dexter, RM Lyons, V Nadar, K Darrow, V Hardman, K Krell, J Rehm, S Rathbun, A Spyropoulos, S Moll, D Chen, D Banish, S Joseph, G Rodgers, S Stevens, P Wright, M Ramaswamy, W Botnick, C Albrecht, A Jaffer, M Kennedy, W Rodriguez-Cintron, N Ettinger, J Gleeson, R Lavender, K Stevens.
